# Supplementary material for: Complexome profiling of the Chlamydomonas psb28 mutant reveals TEF5 as an early PSII assembly factor
Source: Plant Cell. 2025 Mar 17;37(6):koaf055. doi: 10.1093/plcell/koaf055 (PMC12164590; doi:10.1093/plcell/koaf055)
Supplement: koaf055_Supplementary_Data [file koaf055_supplementary_data.zip › psb28_interactive.zip!/plots/intensity/qualityPlots/peptides/ycf3_Cre-1.g2717008.t1.p1.html]

### Cre-1.g2717008.t1.p1

Trivial name: ycf3  
SeqCoverage [median]: 62.20  
Mass: 19.81 kDa  
  
 

| Sequence | MissedCleavage | Mass [Da] | wtIntensity | muIntensity | Score |
| --- | --- | --- | --- | --- | --- |
| AADYWKEAIR | 1 | 1221.61 | 454.22 | 0.00 | 60.52 |
| ALEYYYQALER | 0 | 1417.69 | 14309.35 | 42381.84 | 177.78 |
| EKQAFSYYR | 1 | 1190.57 | 2704.19 | 594.90 | 93.11 |
| GEQAIENGQSEISQILFEK | 0 | 2119.04 | 559.75 | 0.00 | 43.12 |
| LEVDAYDR | 0 | 979.46 | 2411.99 | 13928.66 | 106.36 |
| LTGLAT | 0 | 574.33 | 0.00 | 2544.06 | 76.55 |
| NPSLSSALNNIAVIYHYR | 0 | 2031.05 | 3799.55 | 0.00 | 155.10 |
| QAFSYYR | 0 | 933.43 | 0.00 | 2458.02 | 96.51 |
| SYILYNIGLIHTSNGEHGR | 0 | 2143.08 | 0.00 | 4628.05 | 107.57 |
| VLPTSQR | 0 | 799.46 | 6555.36 | 0.00 | 86.09 |
| VLPTSQREK | 1 | 1056.59 | 501.40 | 1132.08 | 82.29 |
|  |  |  | 31295.8 | 67667.6 |  |
